# Strategies to increase living kidney donation: a retrospective cohort study

**DOI:** 10.1186/s40697-015-0049-6

**Published:** 2015-04-21

**Authors:** Héloïse Cardinal, Céline Durand, Sandra Larrivée, Jacobien Verhave, Michel R Pâquet, Marie-Chantal Fortin

**Affiliations:** Centre de recherche du Centre hospitalier de l’Université de Montréal (CHUM), Montréal, Canada; Nephrology and Transplantation Division, Centre hospitalier de l’Université de Montréal (CHUM), 1560 Sherbrooke Street East, H2L 4M1 Montreal, QC Canada; Bioethics Program, Department of Social and Preventive Medicine, École de santé publique de l’Université de Montréal, Montréal, Canada

## Abstract

**Background:**

Living kidney transplantation (LKT) offers the best medical outcomes for organ recipients. Historically, our centre had a low rate of LKT. In 2009, in an effort to increase living organ donation (LOD), a dedicated team was created. Its mandate was to promote LOD at our centre and at referring centres, to coordinate assessments of living organ donors, to facilitate the process, and to ensure long-term follow-up after the donation. In November 2010, our centre joined the national living donor paired exchange registry (LDPE).

**Objective:**

To document the impact of the LOD team and LDPE registry on LOD rates at our centre.

**Design:**

Retrospective cohort study

**Setting:**

Single center study in a university hospital with an adult kidney transplant program

**Patients:**

Using our electronic database, we included all potential living organ donors who contacted our centre from 01/01/2005 to 31/12/2008 and from 01/01/2009 to 31/12/2012. Follow-up was conducted until 31/12/2013.

**Measurements:**

Number of transplantations from living donors, number of potential donors who contacted the centre, donor and recipient characteristics.

**Methods:**

We compared the number of transplantations from living donors performed and the number of potential donors who contacted the centre before and after the creation of the LOD team and participation in the LDPE.

**Results:**

A total of 50 renal transplantations were performed using organs from living donors during the first time period, whereas this increased to 73 in the 2009-2012 cohort (incidence rate difference (IRD): 0.030, 95% confidence interval (CI) 0.003-0.056). We also observed a significant increase in the number of individuals who contacted our centre to donate a kidney. During the 2005–2008 period (cohort 1), 191 individuals interested in donating a kidney contacted our centre, whereas this figure was 304 during the 2009–2012 period (cohort 2) (IRD: 0.143, 95% CI 0.091-0.196).

**Limitations:**

Single center study, relatively low sample size

**Conclusion:**

The implementation of a LOD team, combined with our participation in the LDPE registry, was associated with a significant increase in the actual number of living kidney transplantations performed. These data support initiatives such as the creation of dedicated LOD teams and LDPE registry to increase LKT.

## Abrégé

**Contexte:**

Le meilleur traitement pour les patients atteints d’insuffisance rénale est la transplantation rénale à partir d’un donneur vivant (DV). Historiquement, notre centre hospitalier réalisait une faible proportion de greffes rénales à partir de DV. En 2009, nous avons créé une équipe dédiée au don vivant afin d’augmenter le nombre de transplantations rénales à partir de DV. Cette équipe avait pour mandat de promouvoir le don vivant dans notre centre et dans les centres périphériques, de coordonner et faciliter l’évaluation des DV et s’assurer d’un suivi à long terme des DV. A partir de novembre 2010, notre centre s’est joint au registre canadien de donneurs vivants jumelés par échange de bénéficiaires (DVEB).

**Objectif:**

Évaluer l’impact de l’équipe dédiée au don vivant et de la participation au registre canadien de DVEB sur le nombre de donneurs potentiels ayant contacté notre centre et le nombre de transplantation effectuées à partir de DV.

**Type d’étude:**

Etude de cohorte rétrospective

**Lieu de l’étude:**

Centre de santé universitaire offrant un programme de transplantation rénale adulte

**Patients:**

Tous les DV qui ont contacté notre centre entre le 01/01/2005 et le 31/12/2008 et entre le 01/01/2009 et le 31/12/2012. Nous avons effectué un suivi jusqu’au 31/12/2013.

**Mesures:**

Nombre de transplantations effectuées à partir de DV, nombre de donneurs potentiels ayant contacté notre centre, caractéristiques des donneurs et des receveurs

**Méthodologie:**

Nous avons comparé le nombre de transplantations à partir de DV ainsi que le nombre de donneurs potentiels qui ont contacté notre centre avant et après la création d’une équipe dédiée au don vivant et la participation au registre de DVEB.

**Résultats:**

Nous avons réalisé 50 transplantations à partir de DV dans la première période, et 73 dans la deuxième (différence du taux d’incidence (DTI) : 0.030, intervalle de confiance de 95% (IC) 0.003-0.056). Entre 2005 et 2008, 191 individus ont manifesté un intérêt à être DV en contactant notre centre, alors que ce chiffre a augmenté à 304 pour la période 2009-2012 (DTI: 0.143, IC 95% 0.091-0.196).

**Limites:**

Étude avec un petit nombre de patients, provenant d’un seul centre de santé

**Conclusion:**

La création d’une équipe de DV et la participation au registre canadien de DVEB sont associés à un accroissement du nombre de donneurs vivants effectifs. Ces données supportent l’efficacité d’initiatives telles la mise en place d’équipes dédiées au don vivant et le registre canadien de DVEB afin d’augmenter le nombre de transplantations à partir de DV.

## What was known before

Given the negative impact of waiting time on dialysis on patient outcomes, finding ways to increase organ supplies for kidney transplantation is crucial. The rate of living kidney donation is lower in the province of Quebec compared to the rest of Canada.

## What this adds

We have observed a significant increase in the number of living kidney transplantations performed at the Centre Hospitalier de l’Université de Montréal after the implementation of a dedicated living donor team and participation in the Canadian National Living Donor Paired Exchange registry, suggesting that these initiatives may have had a beneficial effect on the rate of living kidney transplantation.

## Background

Living kidney transplantation is the best treatment for end-stage renal disease patients [1]. Rates of living kidney transplantation (LKT) vary greatly throughout the world. For instance, in 2011, 34% of renal transplantations in the US were performed using living donor kidneys, compared to only 12% in Spain and 10% in France [2-4]. In Canada, there is a marked difference between rates of living organ donation in Quebec (22%) and the other provinces, where the rates fluctuate between 35 and 52% [5,6]. Factors that might partly explain this difference are higher rates of deceased organ donation and historically shorter wait times in Quebec compared to the rest of Canada. Even so, current waiting time for a kidney transplant in Quebec is over 3 years [5]. Given the negative impact of time on dialysis on patient and graft survival [7], increasing the number of organs available through living donation is crucial.

Historically, our centre (Centre Hospitalier de l’Université de Montréal (CHUM)) has had a low rate of LKT (between 10% and 20% of all renal transplantations performed) [8]. In January 2009, in an effort to increase living organ donation (LOD), a dedicated multidisciplinary team was created. Furthermore, starting in November 2010, our centre joined the Canadian National Living Donor Paired Exchange (LDPE) registry, making possible transplantations that would otherwise not have been performed in the context of ABO incompatibility or crossmatch positivity between donor-recipient pairs. In this study, we aimed to assess whether the implementation of these initiatives (living donor team, LDPE registry) was associated with differences in the number of LKT performed, the number and conversion rate of potential donors who contacted our centre, and the characteristics of these actual and potential donors.

## Methods

### Study design, subjects and data collection

We performed a retrospective cohort study of potential and actual living donors who contacted the CHUM between 2005 and 2012. All potential living donors who contacted the CHUM from 01/01/2005 to 31/12/2008 (cohort 1) and from 01/01/2009 to 31/12/2012 (cohort 2) were included in this study. Follow-up was conducted until 31/12/2013. All the individuals who contacted our centre and talked to the coordinator or the nurse about donating an organ were included in the analysis, even if the process went no further than this phone call. During the call, the coordinator conducted a screening interview. If there were no obvious medical contraindications, the process was continued. A medical questionnaire was sent to the potential donor. Once the completed questionnaire was returned, an appointment was scheduled with the nurse and the nephrologist responsible for LOD. There was no change in acceptance criteria for donation during the study periods. All these data were captured in our transplantation database. This research project was approved by the CHUM’s research ethics board.

### Intervention

Before 2009, patients who were referred to our center received information on the option of LOD at the time they were evaluated for eligibility for kidney transplantation. Patients were instructed to ask their potential donors to contact the transplant centre if they were willing to undergo evaluation for LOD.

In January 2009, we created a multidisciplinary team whose purpose was to promote LOD. This dedicated team included a transplant nephrologist, two transplant surgeons, one nurse, one transplant coordinator, two psychologists and one social worker. One of its mandates was to promote LOD at our centre and referring centres. First, this team gave presentations on living kidney donation and transplantation in all the CHUM’s referral hemodialysis centres. The presentations were given on weekday evenings, on the referral centre’s site, by a team of 2 transplant nurses, a transplant nephrologist and a transplant surgeon. The presentations lasted 2 hours and covered the following themes: benefits of kidney transplantation versus dialysis, benefits of living kidney transplantation compared to deceased donor transplantation, surgical procedures for the donor and recipient, immediate and long-term complications for the donor and recipient. Patients, families and healthcare professionals were invited to attend these presentations. The other mandates of this team were to coordinate assessments of living organ donors, to facilitate the process, and to ensure long-term follow-up after the donation. In November 2010, the province of Quebec joined the Canadian national LDPE registry.

### Outcomes

The primary outcome was the difference in the number of LKT performed before and after the implementation of these interventions. Secondary outcomes were the differences in the number of potential donors who contacted our center, their conversion rate to actual donation, and their characteristics before and after the implementation of the living donor team.

### Statistical analyses

The following data were retrieved from electronic database: age, gender and blood group of potential donors and recipients; relationship between donors and recipients; outcomes (refusal, transplantation, etc.); the recipient’s renal replacement therapy; and the time between the initial contact and the donation. Continuous variables are summarized with means and standard deviations (SD), and categorical variables are summarized using proportions. We used Student *T* tests to compare differences in continuous variables and chi-square tests for differences in proportions between cohorts 1 and 2. Finally, incidence rates for transplantations performed with living donors and the number of potential living donors who contacted our center were calculated by dividing these events by the person-time of all patients who were registered and active in our kidney transplant waiting list in each period. All transplantations were classified in the period that corresponds to the date of the initial contact by the donor. Incidence rate differences (IRD) and 95% confidence intervals (CI) are reported. Statistical analyses were performed using SPSS software, version 21 (IBM).

## Results

### Effective donors

There was a significant increase in the number of LKT performed at our center when cohort 1 and 2 were compared (cohort 1: 50 in 844.94 person-years of follow-up, cohort 2: 73 in 823.47 person-years of follow-up, (IRD: 0.029, 95% CI 0.003-0.056). In contrast, the number of transplantations originating from deceased donors remained similar (201 in 844.94 person-years between 2005 and 2008 and 183 in 823.47 person-years between 2009 and 2012, IRD: −0.016, 95% CI −0.062-0.030). During the two periods, the total number of LKT performed in the province of Quebec remained similar (190 between 2005 and 2008 and 181 between 2009–2012). The recipients’ characteristics were similar in both cohorts (Table 1). There were 2 pediatric transplants in cohort 1 and six in cohort 2 (p = NS). Nine transplantations were performed through the LDPE program in period 2.

**Table 1:** **Characteristics of kidney transplant recipients**

	**Cohort 1 (2005–2008) n = 50 (%)**	Cohort 2 (2009–2012) n = 73* (%)	**p-value**
Male gender (%)	29 (58.0)	41 (56.9)	NS
Blood group (%)			NS
A	21 (42.0)	31 (43.1)	
B	3 (6.0)	9 (12.5)	
AB	3 (6.0)	2 (2.7)	
O	23 (46.0)	30 (41.7)	
Age in years (±SD†)	41.4 ± 11.8	41.4 ± 15.5	NS
Number of pairs with a group O donor and a non group O recipient	8 (16.0)	18 (24.7)	NS
Renal replacement therapy (%)			NS
Hemodialysis	28 (56.0)	37 (51.4)	
Peritoneal dialysis	7 (14.0)	8 (11.1)	
None	15 (30.0)	26 (36.1)	
Unknown	0 (0)	1 (1.4)	
Pediatric transplants	2 (4.0)	6 (8.3)	NS

*There was missing data for 1 recipient.

†SD: standard deviation.

Among the effective donors, there were more women than men, and a predominance of blood type O donors in both groups. With the exception of one altruistic donor in cohort 2, all actual donors were either genetically or emotionally related to their recipients. In cohort 1, 70% of donors were first degree family members of the potential recipient (parent, children and sibling), but this proportion decreased to 50.7% in cohort 2 (p = 0.03). Table 2 summarizes the data for effective donors. The proportion of donors who experienced a delay in the donation process, defined as a period of >300 days between first contact and organ procurement, was higher in cohort 2 (p = 0.002). Aside from participation in the LDPE program, we could not identify particular reasons explaining this difference (Table 3).

**Table 2:** **Characteristics of actual donors**

	**Cohort 1 (2005–2008) n = 50 (%)**	**Cohort 2 (2009–2012) n = 73 (%)**	**p-value**
Male gender (%)	20 (40.0)	34 (46.6)	NS
Blood group			NS
A	18 (36.0)	22 (30.1)	
B	1 (2.0)	3 (4.1)	
O	31 (62.0)	48 (65.8)	
AB	0 (0)	0 (0)	
Age in years (±SD*)	45.6 ± 8.8	47.3 ± 11.2	NS
First degree relatives † (%)	35 (70.0)	37 (50.7)	0.033
Delay in the donation process § (%)	19 (38.0)	48 (65.8)	0.002

*SD: standard deviation.

†First degree relative defined as parent, child or sibling.

§delay defined as >300 days elapsed between first contact and organ procurement.

**Table 3:** **Cause of delays (>300 days elapsed between first contact and organ procurement) in the donation process**

	**Cohort 1 (2005–2008) n = 19**	**Cohort 2 (2009–2012) n = 48**	**p-value**
Participation in the LDPE* program	0 (0)	9 (12.3)	0.010
Donor or recipient medical condition†	16 (13.0)	28 (22.8)	0.47
Logistic considerations§	3 (6)	11 (23)	0.12

*LDPE Living Donor Paired Exchange.

†Donor conditions included need for kidney biopsy, smoking cessation, weight loss, hypertension, investigation for renal cysts, psychological consultation; recipient conditions included infections, diverticulitis, need for cholecystectomy or partial colectomy, further cardiac evaluation, and investigation for an ovarian cyst.

§Logistic considerations included foreign donors, moves, separations, travelling, and work schedule considerations.

### All potential donors

We observed an increase in the number of potential donors who contacted our centre in the period that followed implementation of the LOD team (cohort 1: 191 in 844.94 person-years of follow-up, cohort 2: 304 in 823.47 person-years of follow-up (IRD: 0.143, 95% CI 0.091-0.196). Again, the type of relationship between potential donors and recipients differed between the two cohorts. In 59.2% of cases in cohort 1 and 43.4% in cohort 2, the potential donor was a first degree relative of the recipient (p < 0.05). Cohort 2 comprised 13 potential altruistic donors, while none were observed in cohort 1. Table 4 summarizes the data for potential donors.

**Table 4:** **Characteristics of all potential donors**

	**Cohort 1 (2005–2008) n = 191**	**Cohort 2 (2009–2012) n = 304**	**p-value**
Conversion from potential to actual donation (%)	50 (26.2)	73 (24.0)	NS
Male gender* (%)	81 (42.4)	120 (39.5)	NS
Blood group* (%)			
A	54 (28.3)	93 (30.6)	NS
B	10 (5.2)	23 (7.6)	
O	101 (52.9)	152 (50)	
AB	1 (0.5)	5 (1.6)	
Age in years (±SD†)	46.4 ± 11.3	47.7 ± 12.6	NS
First-degree relatives (%)	113 (59.2)	132 (43.4)	0.001

*Missing data on 1 potential donor’s gender and on 56 potential donor’s blood group.

† SD standard deviation.

### The non-donors

**Figure 1:**
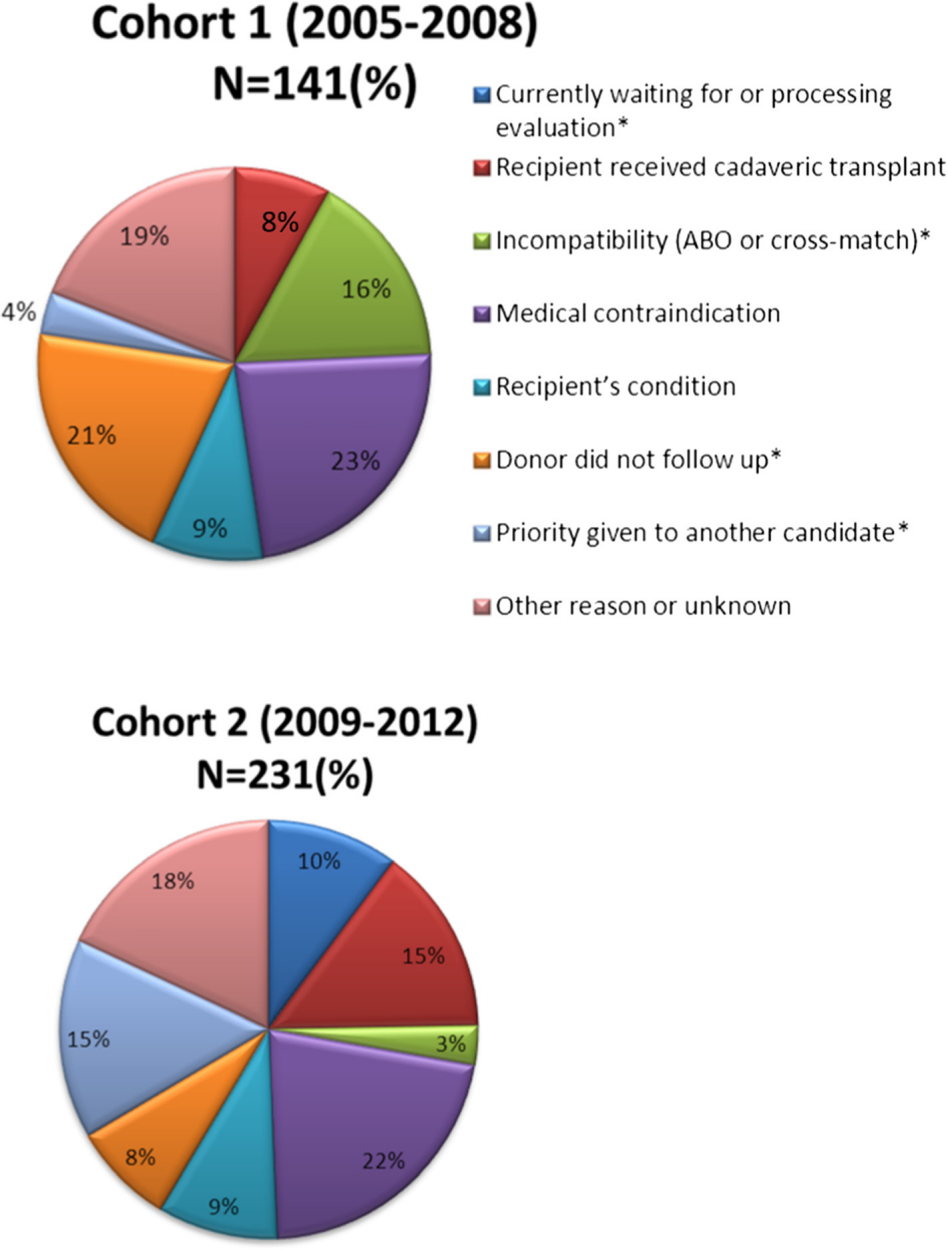
Motives explaining failure to donate in cohort 1 and 2. *The reasons for which potential donors did not actually donate differed between period 1 and 2 in terms of ABO or crossmatch incompatibility (16% in cohort 1 versus 3% in cohort 2, p < 0.001), priority given to another donor (4% in cohort 1 versus 15% in cohort 2, p < 0.001), lack of follow-up on the part of the donor (21% in cohort 1 versus 8% in cohort 2, p < 0.001), and an evaluation that is still being processed (0% in cohort 1 versus 10% in cohort 2, p < 0.001).

Approximately 75% of potential living donors did not donate in both cohorts. The rate of refusal by the transplant team for medical reasons was similar between the 2 study periods (23.4% in cohort 1 and 21.2% in cohort 2, p = NS). Termination of the donation process due to a recipient who became unsuitable for transplantation was also similar (9.2% in cohort 1 and 8.2% in cohort 2, p = NS). In both cohorts, a small proportion of potential donors did not donate because their recipient was transplanted with a kidney from a deceased donor (7.8% in cohort 1 and 14.3% in cohort 2, p = 0.06). However, potential donors from cohort 2 were less likely to discontinue the donation process for a lack of interest (ex, by not scheduling appointments) (20.6% in cohort 1 and 7.8% in cohort 2, p < 0.001), and more likely to discontinue the donation process because there were other potential donors for the same recipient who were either being evaluated or found to be a better match for immunologic or other reasons (3.6% in cohort 1 and 15.2% in cohort 2, p < 0.001). In cohort 1, another important reason for not donating was an ABO incompatibility or a positive cross-match with the intended recipient (16.3% in cohort 1 vs. 3% in cohort 2, p < 0.001). Figure 1 summarizes the reasons for termination of the donation process.

## Discussion

The promotion of LOD is extremely important in the face of an ever increasing demand for kidney transplantation. Hence, we were interested in evaluating the local impact of the implementation of a dedicated LOD team and participation in the LDPE registry on LOD rates, and on the number and characteristics of potential donors. In the period that followed the onset of these initiatives, we observed a higher number of actual LKT performed and of potential donors who contacted our centre. Furthermore, there was a difference in the relationship between potential donors and recipients between study periods, as we observed an increase in potential donors who were not first-degree relatives (including altruistic donors) in period 2. The conversion rate of potential to actual donors was similar over the study period. However, the reasons for terminating the donation process were different. Potential donors were less likely to discontinue the process due to lack of motivation, ABO incompatibility or crossmatch positivity, but more likely to terminate the process because their intended recipient had another donor after compared to before the implementation of the LOD team and participation in the LDPE registry.

The increase in LKT between the study periods is not likely to be due to improvements in organizational capacity for kidney transplantation at large in our center, as the number of transplantations originating from deceased donors was stable over the study period. Furthermore, the increase is unlikely to be due to secular trends secondary to media attention or societal changes in the province of Quebec, as the number of LKT performed in the whole province remained stable over that time period. This suggests that the observed increase in LKT observed at our centre could be due to the impact of the dedicated living donor team and participation in the LDPE registry, although the retrospective nature of the study cannot allow us to draw a definite conclusion about causation. The effect we report may also be underestimated as follow-up time was shorter for cohort 2, and since 4 suitable cohort 2 living donors are registered in the LDPE but have not yet been matched.

The LOD team and participation in LDPE could both have contributed to the increase in LKT we observed in 2009–2012 compared to 2005–2008. The dedicated LOD team could have contributed to improved LKT rates mostly through the increase in the number of potential donors who contacted our centre. This could be the result of the presentations on living kidney donation and transplantation given by the team in different nephrology centres where patients and their families and professionals were invited to attend. The dissemination of information and knowledge on living donation through these presentations may also explain the change we noted in the relationship between the donors and their intended recipients over the study periods. Education on the excellent outcomes of LKT in recipients of non-genetically related donors may have led to an increase in the number of contacts from spouses and friends who were willing to donate. One of the goals of the LOD team was to facilitate the living donation process. Hence, team members helped the potential donor to schedule appointments and made sure that the required tests were obtained in a timely fashion. This probably explains why we observed different reasons for terminating the donation process before and after the LOD team became operational. For instance, we noted a decreased proportion of potential donors who did not schedule their appointments in cohort 2. Although this may make the process less cumbersome for potential donors, the time elapsed between first contact and organ procurement was longer in cohort 2 than in cohort 1. Except for participation in the LDPE program, we could not identify specific reasons for this prolongation in cohort 2 versus cohort 1. We observed that 13 potential altruistic donors contacted our centre between 2009 and 2012, whereas none presented themselves between 2005 and 2008. Increased media attention on this type of living donation and an attitude of openness from the transplant professionals could be hypothesized for this finding. However, this cannot explain the increase in LKT we observed, as only one of these potential donors actually resulted in effective donation.

Before the participation of Quebec in the LDPE (November 2010), blood group and immunologic incompatibilities were an important reason to not perform living kidney transplantation [8]. The LDPE has removed this reason for refusal. Also, the LDPE has allowed 9 recipients from our center to receive a LKT. Moreover, four pairs are registered and waiting for a match. These results support the implementation of LDPE. It is also worth noting that a significant proportion of pairs in cohort 2 (24.7% or 18) were composed of an O blood group donor and a non O blood group recipient. These pairs could have been recruited to participate in LDPE in order to decrease the disadvantage of O blood group recipients in LDPE [9].

## Conclusion

In addition to being the best option from the patient perspective, LKT also allows for substantial savings for society: LKT recipient are taken off dialysis and are removed from the deceased donor waiting list, which increases the likelihood of finding a deceased donor for those who have no living donor available [10]. In our transplant program, the implementation of a LOD dedicated team and participation in the LDPE were associated with an increased number of living kidney transplantations performed in recent years. These results support the implementation of such initiatives to promote LOD.
